# Magnetoelastic Monitoring System for Tracking Growth of Human Mesenchymal Stromal Cells

**DOI:** 10.3390/s23041832

**Published:** 2023-02-07

**Authors:** William S. Skinner, Sunny Zhang, Jasmine R. Garcia, Robert E. Guldberg, Keat Ghee Ong

**Affiliations:** Knight Campus for Accelerating Scientific Impact, University of Oregon, Eugene, OR 97403, USA

**Keywords:** magnetoelastic, magnetostrictive, sensor, monitoring, wireless, mesenchymal, stromal, resonance

## Abstract

Magnetoelastic sensors, which undergo mechanical resonance when interrogated with magnetic fields, can be functionalized to measure various physical quantities and chemical/biological analytes by tracking their resonance behaviors. The unique wireless and functionalizable nature of these sensors makes them good candidates for biological sensing applications, from the detection of specific bacteria to tracking force loading inside the human body. In this study, we evaluate the viability of magnetoelastic sensors based on a commercially available magnetoelastic material (Metglas 2826 MB) for wirelessly monitoring the attachment and growth of human mesenchymal stromal cells (hMSCs) in 2D in vitro cell culture. The results indicate that the changes in sensor resonance are linearly correlated with cell quantity. Experiments using a custom-built monitoring system also demonstrated the ability of this technology to collect temporal profiles of cell growth, which could elucidate key stages of cell proliferation based on acute features in the profile. Additionally, there was no observed change in the morphology of cells after they were subjected to magnetic and mechanical stimuli from the monitoring system, indicating that this method for tracking cell growth may have minimal impact on cell quality and potency.

## 1. Introduction

Increases in the success of clinical treatments using stem cells for chronic and historically difficult-to-treat ailments have generated a demand for scalable cell cultures and characterization methods that can consistently produce large quantities of high-quality stem cells [1]. To ensure the quality of cells, cell manufacturing processes often track multiple critical process parameters (CPPs), which are conditions in processes that have a significant impact on the resulting cell quality. In the cell manufacturing community, cell quality is characterized by their critical quality attributes (CQAs), which are established and quantifiable characteristics that can reliably evaluate the therapeutic potential of a cell product. Today, state-of-the-art technologies for monitoring CPPs and characterizing CQAs of anchorage-dependent cells typically require staining or detaching the cells, measuring the DNA content, or analyzing their metabolic activity [2,3,4,5]. The harsh and invasive nature of these methods increases the likelihood of errors in characterization or degradation in cell quality [6]. Furthermore, these manual, labor-intensive methods are difficult to incorporate into automatic feedback-controlled processes; thus, reliance on these methods could hinder the advancement of large-scale manufacturing of stem cells. Other non-invasive methods have been employed in 2D and 3D cell cultures to provide temporal assessment of cell culture, including techniques such as cell-substrate electrical impedance measurements and in situ microscopy and imaging [7,8,9]. However, methods such as electrical impedance or microscopy cannot be readily functionalized to monitor certain CPPs such as glucose concentration and pH, limiting their use in cell manufacturing. In light of these challenges, we have developed a scalable sensor platform for contact-less, longitudinal, in-line monitoring of conditions of adherent cells. The sensor platform is based on magnetoelastic materials, which are sensitive to mechanical loading (i.e., from cell attachment) and generate a response when probed by magnetic fields, facilitating wireless sensing of cells inside the bioreactor. The realization of a scalable platform that can non-invasively provide real-time measurement of cell numbers is an indispensable consideration in the optimization of large-scale, high-volume cell cultures.

**Figure 1:**
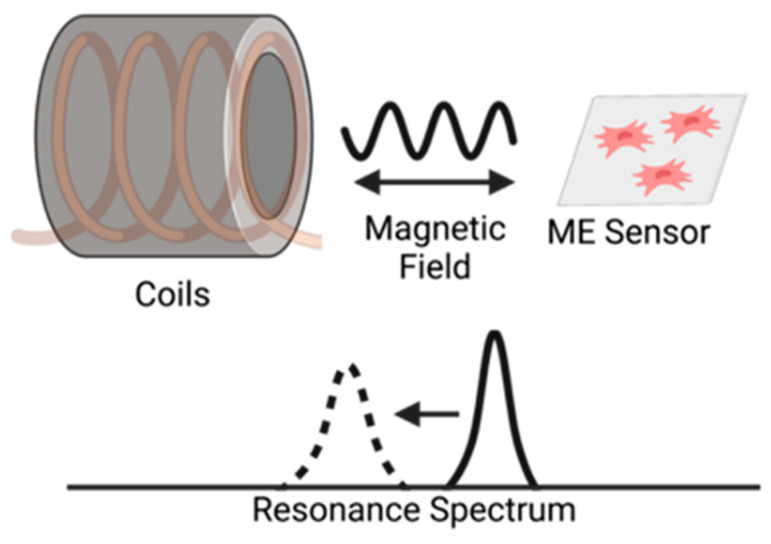
Illustration of the interrogation of a magnetoelastic sensor by measuring the change in its resonance spectrum. The magnetoelastic sensor and detection coil are inductively coupled, and the sensor’s resonance spectrum is recorded by measuring the coil’s electrical impedance spectrum.

Magnetoelastic sensors have many advantages for monitoring cells because they can be scaled into different sizes, coated with biocompatible and functionalized materials, and be activated and interrogated remotely [10,11]. The sensing capability of a magnetoelastic sensor is based on its magnetostrictive property, which allows it to physically vibrate and generate a secondary magnetic flux at its resonance frequency when exposed to a frequency-varying alternating magnetic field (Figure 1). As mass (cells) accumulates on the surface of the sensor, the resonance characteristics, including the resonance quality, resonance frequency, and magnitude of resonance, reduce in value. The changes in these resonance characteristics can be used to determine cell count in the bioreactor and other CPPs. Magnetoelastic sensors have been successfully used in the past to monitor biological environments and markers; for example, multiple groups have employed the magnetoelastic sensors as immunosensors to detect and identify bacterial populations of *Staphylococcus aureus* and *Escerichia coli* [12,13], as well as other biological agents, pathogens, and endotoxins [14,15,16,17,18,19,20]. Magnetoelastic sensors have also been implemented in the monitoring and quantification of breast cancer cell (MCF-7) growth [21]. Recently, magnetoelastic sensors were explored for use in cell manufacturing processes. For example, our previous work demonstrated that magnetoelastic sensors can be treated to improve biocompatibility and cytocompatibility with fibroblast cells and to modulate cell properties and adhesion [22,23,24,25], as well as longitudinally track cell number of fibroblasts seeded on the surface of these sensors [26].

In this study, we evaluated the viability of our monitoring system for use with human mesenchymal stromal cells (hMSCs). MSCs account for roughly ten percent of the stem cell therapeutic market [27]; thus, the use of this technology to track CQAs and CPPs in hMSC manufacturing could be beneficial to stem-cell based therapies by improving the yield and quality of cells produced. Specifically, we evaluated the sensitivity of the detection system and its ability to capture a temporal profile of hMSC growth, as previously demonstrated with L929 fibroblasts [26]. Additionally, we evaluated the effects of a gelatin coating on the sensor surface and investigated the effect of mechanical and magnetic field stimulus on the cell morphology.

## 2. Materials and Methods

### 2.1. Sensor Fabrication

**Figure 2:**
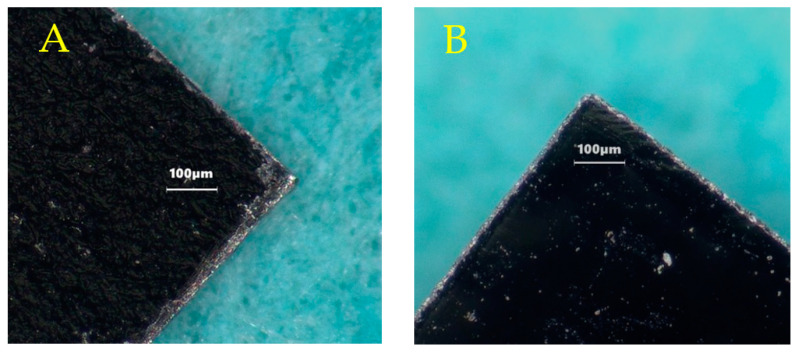
Microscopic images of a corner of mechanically sheared and annealed sensor (**A**), left; compared with a laser-cut sensor (**B**), right. No major differences in resonance behavior were observed between the two methods of sensor fabrication (see Figure 3).

**Figure 3:**
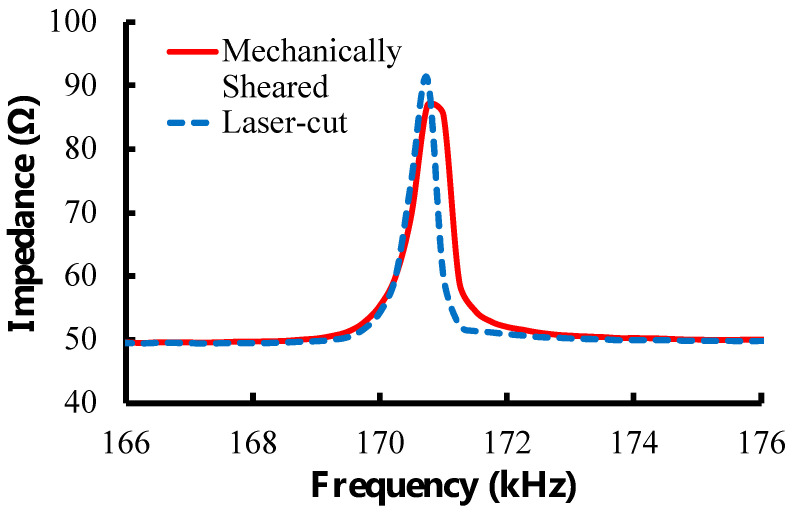
Characteristic resonance spectra for laser-cut and mechanically sheared sensors. The sensors share similar resonance frequencies and magnitude. The laser-cut sensor has a more defined peak and higher quality factor than the mechanically sheared sensor, making laser cutting the preferred method to manufacture sensors.

Sensors were made from a commercially available magnetoelastic material, Metglas 2826 MB (Metglas Inc., Conway, SC, USA) [28]. Metglas 2826 MB was used because of its good magnetization (saturation magnetization = 0.39 T) and low coercivity (coercive force < 50 A/m) that result in strong signals as well as large magnetostriction (saturation magnetostriction = 12 ppm) that makes them sensitive to mechanical loading. The material was purchased as a rolled, thick-film strip of 12.7 mm wide and 29 μm thick. A stack of 4 magnetoelastic strips of about 15 cm long was placed on top of a 0.5 mm thick steel sheet (McMaster-Carr, Elmhurst, IL, USA) that was covered in a layer of aluminum foil (Thermo Fisher Scientific, Waltham, MA, USA). Rectangular sensors of 0.5 × 1.27 cm were cut from the strips with a high-power pulse laser (Coherent ExactCut 430, Santa Clara, CA, USA) operating with a frequency of 2000 Hz, 0.05 ms pulse width, 600.0 W peak power and 60.00 W average power. Compared to the mechanically sheared and annealed sensors (average resonance frequency = 170.03 ± 0.90 kHz; *n* = 10) used in previous experiments [26,29], these laser-cut sensors (average resonance frequency = 170.15 ± 0.43 kHz; *n* = 10) were found to have similar physical appearance and performance; thus, the annealing step was omitted here (Figure 2 and Figure 3). The laser-cut sensors were coated with a 10 µm Parylene-C conformal layer via a commercially available vapor deposition coating system (PDS 2010 Labcoater, SCS, Indianapolis, IN, USA). The coated sensors were then treated with oxygen plasma in a reactive etching system (March Jupiter II RIE system, Nordson March, Concord, CA, USA) for 30 s at 100 W. This coating technique was developed by Holmes et al. and employed in the fabrication of sensors for the detection of L929 fibroblast cells [22,26].

### 2.2. Sensor Stage Fabrication

**Figure 4:**
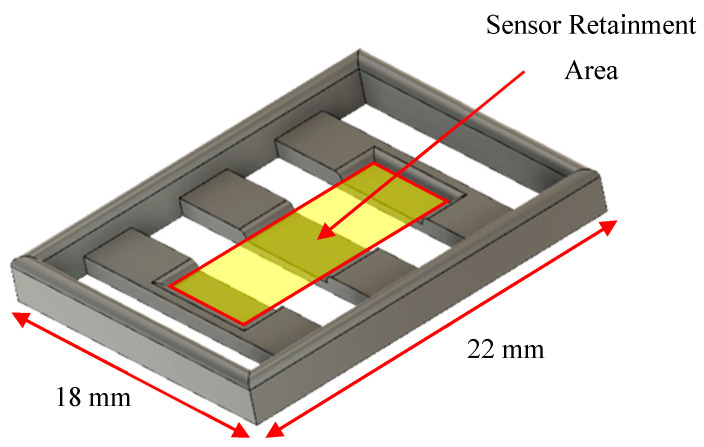
CAD drawing of a stage designed to retain and suspend the sensor in the chamber slide.

Sensors were suspended in wells by a grated stage with a recessed retainment area (Figure 4). Sensor stages were designed with computer-aided design (CAD) (Autodesk Fusion 360 v2.0) and 3D printed using a stereolithography (SLA) printer (Formlabs, Somerville, MA, USA) with biocompatible and autoclavable resin (High Temp V2 Photopolymer Resin, Formlabs, Somerville, MA, USA).

### 2.3. Detection System

**Figure 5:**
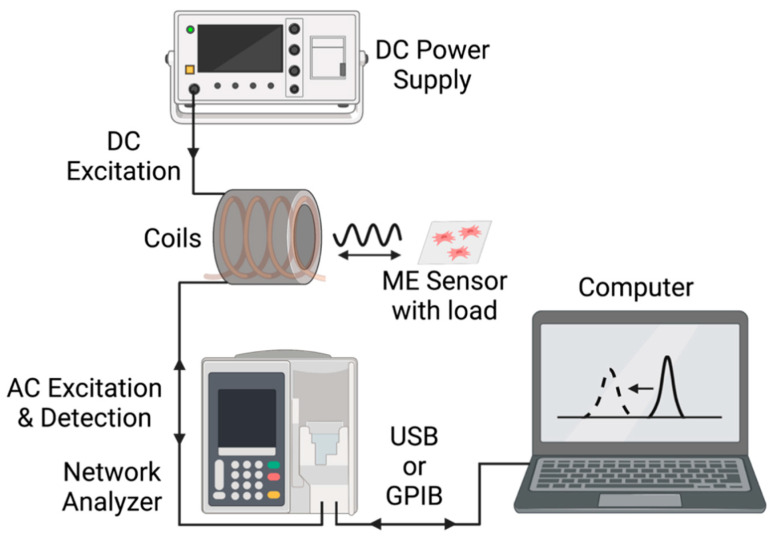
Illustration of the key equipment and interfaces in this experimental setup.

As illustrated in Figure 5, the magnetoelastic sensors were interrogated with a custom detection system consisting of two concentric wound solenoids. The external solenoid was connected to a DC power source that supplied a DC current at 1.2 A, resulting in the generation of a DC magnetic field calculated to be 1.7 kA/m. Previous research determined that 1.7 kA/m was the optimal DC bias field for the sensors of this size and detection system used in this experiment [29]. The internal solenoid, wound with 32-gauge magnet wire, was connected to a network analyzer (Keysight ENA Network Analyzer E50618, Keysight Technologies, Santa Rosa, CA, USA and NanoVNA-F, Hangzhou Minghong Electronic Technology Co., Ltd., Hangzhou, China), which captured the resonance from the sensors by measuring the spectrum of impedance at the detection coil’s terminal. The resonance of the sensor appeared as a peak in the impedance spectrum. As mechanical force/stress was applied to the sensor, the measured resonance characteristics, including the magnitude, quality factor, and resonance frequency of the peak, changed. Tracking one of more of these characteristics allows for the measurement of the mechanical force/stress.

**Figure 6:**
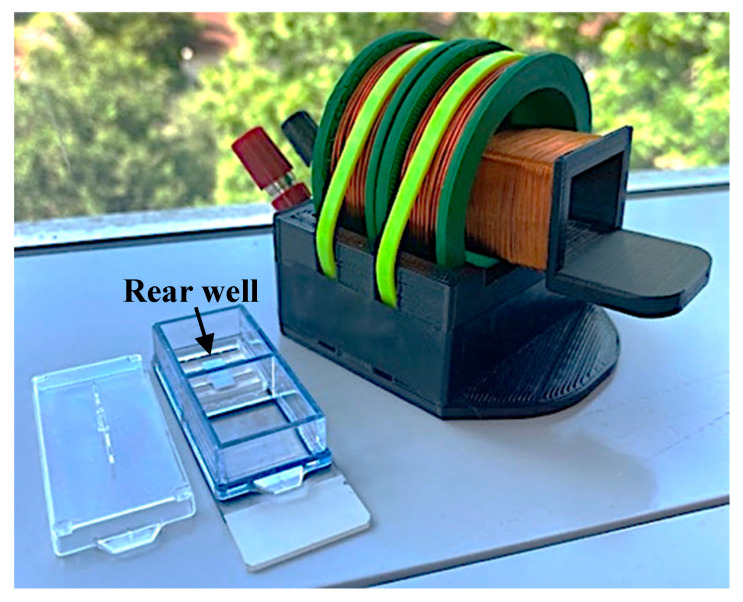
Photograph featuring the placement of the sensor and stage inside the chamber slide well. The internal coil is pulled partially out of the external coil to show detail in this image.

The network analyzer and the connecting cable to the detection coils were calibrated together with a manual open-short-load calibration kit (Hangzhou Minghong Electronic Technology Co., Ltd., Hangzhou, China). Following calibration of the cable and network analyzer, the coils were connected, and the impedance spectra of the coils were recorded prior to placement of the sensors as a background measurement. Next, a single sensor was placed in the rear well (Figure 6) of a 2-well chamber slide (Nunc Lab-Tek II Chamber Slide, Thermo Fisher Scientific, Waltham, MA, USA) on top of the sensor stage so that the sensor can vibrate freely while remaining at the same position. The chamber slide was placed inside the detection solenoid to record the resonance spectrum of the sensor inside. The background measurement was subtracted from all sensor measurements to eliminate the impedance of the coil from the collected data.

### 2.4. hMSC Cell Culture

Bone marrow-derived hMSCs were obtained (donor #000175, female, 28 years old, RoosterBio, Frederick, MD, USA), expanded, and cryogenically stored in liquid nitrogen. hMSCs (PDL 13) were recovered from liquid nitrogen, thawed, and seeded at 5000 cells/cm^2^ until confluent. The cells were cultured in xeno-free media (RoosterBio, Frederick, MD, USA) supplemented with 1% penicillin–streptomycin (Sigma-Aldrich, St. Louis, MO, USA) and xeno-free supplement (RoosterBio, Frederick, MD, USA). Once confluent (~72 h, confirmed via light microscopy), the cells were lifted with 0.25% trypsin-EDTA (Gibco, Invitrogen, Waltham, MA, USA), counted using an automatic cell counter (NucleoCounter NC-200, Chemometec, La Jolla, CA, USA) and then diluted with media to the target seeding density. Cells were incubated at 37.0 °C, ~93% relative humidity, and under 5% CO_2_ in a microbiological incubator (HERAcell VIOS 160i, Thermo Fisher Scientific, Waltham, MA, USA).

### 2.5. hMSC Cell Seeding

Fabricated sensors were cleaned with 70% ethanol, allowed to dry, placed inside sterilization pouches and then sterilized with ethylene oxide gas. Fabricated sensor stages were cleaned with 70% ethanol in a sonicator (CO-Z Ultrasonic Cleaner, CO-Z supplies, Lake Forest, CA, USA) for 5 min, placed inside sterilization pouches, and autoclaved (Beta Star Small Sterilizer Autoclave, Beta Star, Honey Brook, PA, USA). Sterilized sensors were placed in sterile culture dishes, covered with 0.1% gelatin solution (EmbryoMax ultrapure water with 0.1% gelatin, MilliporeSigma, Burlington, MA, USA), and placed in the incubator for 30 min unless otherwise stated. Sterilized sensor stages were placed inside the rear well of a two-well chamber slide (see Figure 6). Sensors were then removed from the gelatin solution and placed inside the sensor retainment area of the sensor stage inside the two-well chamber slide. Cells were seeded at densities ranging from 10,000 to 40,000 cells/cm^2^.

### 2.6. Sensitivity Curve

Chamber slides containing sensors seeded with cells were incubated for 30 min before the first (0 h) resonance spectrum was collected. The resonance spectrum was collected by placing the portion of the chamber slide containing the sensor within the detection solenoid connected to the network analyzer (Keysight ENA Network Analyzer E50618, Santa Clara, CA, USA) and by measuring the impedance from 150 to 200 kHz. The position of the chamber slide was adjusted while monitoring the resonance amplitude of the sensor until the maximum peak magnitude was detected for each sensor. Using the same technique, a second resonance spectrum was collected for each sensor 24 h after seeding.

### 2.7. Longitudinal Tracking of Cell Loading

A detection solenoid was cleaned thoroughly with 70% ethanol and 10% household bleach before placing it inside of an incubator. Chamber slides containing sensors seeded with cells were incubated for 30 min prior to their placement inside the detection coil. The detection coil was connected to a network analyzer (NanoVNA-F) controlled by custom Visual C# software programmed onto a handheld computer (Raspberry Pi 4B, Raspberry Pi Foundation, Cambridge, UK). Resonance measurements were collected once per hour by taking the average of 5 measurements captured in rapid succession.

### 2.8. Staining, Imaging, and Counting of Cells on Sensors

**Figure 7:**
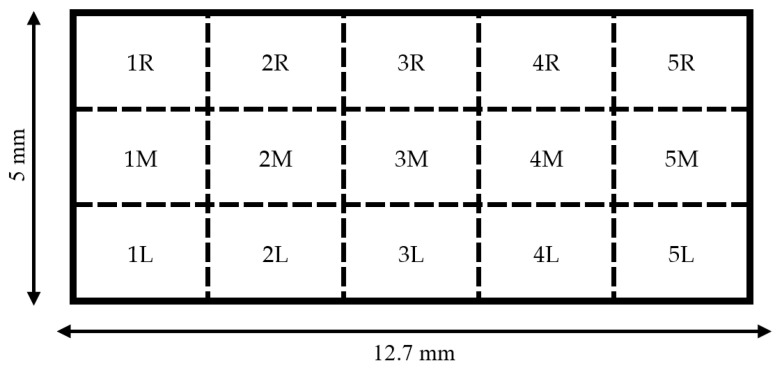
Diagram of regions that were targeted for imaging on each sensor. A 1 mm × 1 mm image was collected at the center for each region for a total of 15 images per sample.

Following each experimental procedure, culture media were aspirated from the well containing the sensor. Next, the well was rinsed with phosphate-buffered saline (PBS) (Endotoxin-Free Dulbecco’s PBS (1X) (w/o Ca^++^ and Mg^++^), Millipore Sigma, Burlington, MA, USA). The PBS rinse was aspirated, and the cells were fixed by incubating the sensors in a solution of 4% paraformaldehyde (PFA) (32% paraformaldehyde aqueous solution, Electron Microscopy Sciences, Hatfield, PA, USA) diluted in PBS for 10 min. Following fixation, PFA solution was aspirated, and the samples were protected from light by wrapping the chamber slides in aluminum foil. Sensors were stained by covering with a solution of Hoescht nuclei stain (Hoescht 33342, Trihydrochloride, Trihydrate, Thermo Fisher, Waltham, MA, USA) diluted to 50 μg/mL in PBS and incubated for 10 min. Following the incubation period, the staining solution was aspirated, and forceps were used to carefully invert the sensor via the stage into the empty, unused well of the chamber slide. The stage was removed, the sensor was covered with PBS, wrapped in foil, and imaged immediately. Fluorescent microscopic imaging was performed using a confocal fluorescence microscope (CSU-W1 SoRa, Nikon, Minato City, Tokyo, Japan) equipped with a 10× magnification lens. Fifteen 1 mm × 1 mm images of the sensor surface were taken at specific locations that were kept consistent for each sensor (Figure 7). Cell nuclei were quantified by manually marking and counting the cells via ImageJ software v1.53t.

## 3. Results and Discussion

### 3.1. Heterogeneity of Cell Distribution on Sensors

**Figure 8:**
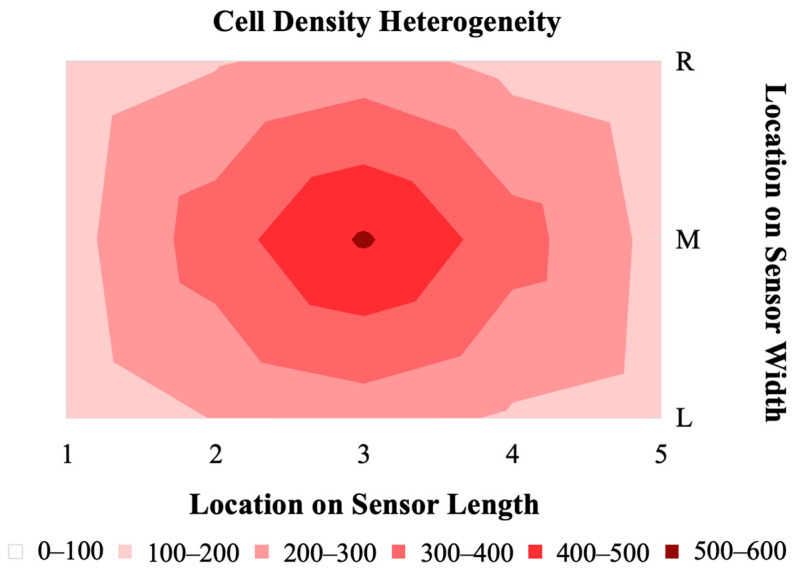
A 2D map representing the heterogeneity in cell density across the sensor surface after 24 h of growth and following resonance measurements at 0 and 24 h (*n* = 5).

The distribution of hMSCs on magnetoelastic sensors formed a consistent pattern of the highest cell density at the center of the sensor, with a decreasing gradient toward the edges (Figure 8). This gradient distribution of cells is the result of a short period of growth (24 h), the chosen seeding density, and the colony-forming behavior of hMSCs. Research by Neuhuber et al. demonstrated that the plating (seeding) density of MSCs has an effect on the growth pattern and kinetics of the cells [30]. The group observed that cultures seeded at high density were evenly spread, where cultures seeded at lower densities grew in colonies. In the case of our sensor, the seeding conditions and duration of culture facilitated the consistent formation of a colony in the center of the sensor. Selecting a higher seeding density or increasing the duration for which the cells are allowed to grow could result in a more homogenous distribution of cells on the sensor surface. There were no observed differences in cell distribution gradient between the resonated sensors and non-resonated control sensors, indicating that the vibrations due to the sensor resonance did not contribute to the cell density distribution.

### 3.2. Evaluation of Sensor Sensitivity

**Figure 9:**
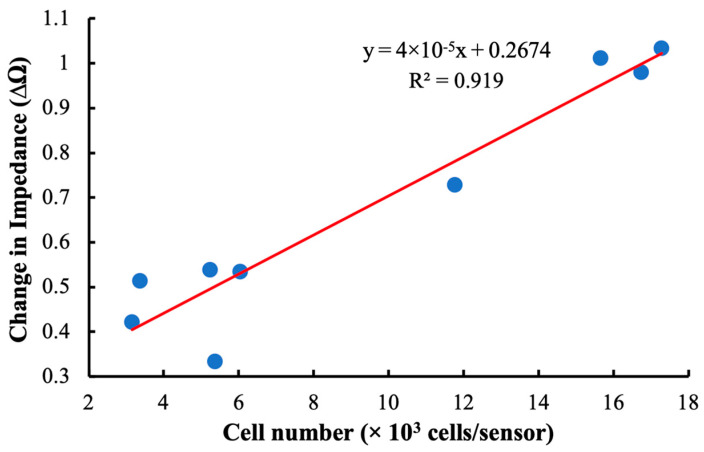
A curve comparing the shift in impedance at resonance frequency of the sensor to the total number of cells attached to the sensor surface. The linear relationship indicates that there is a direct relationship between changes in impedance at resonance and cell loading at the sensor surface.

As shown in Figure 9, our detection system was sensitive to hMSCs loaded at densities between 3000 and 18,000 cells/cm^2^. The linearity of the plot indicates that there was a direct relationship between the total number of cells attached to the sensor surface and changes in the impedance at resonance. The relatively high coefficient of determination indicates that this relationship is robust and that the monitoring system is a good candidate for further development and implementation in larger cell-culture systems.

### 3.3. Longitudinal Tracking of Cell Loading

**Figure 10:**
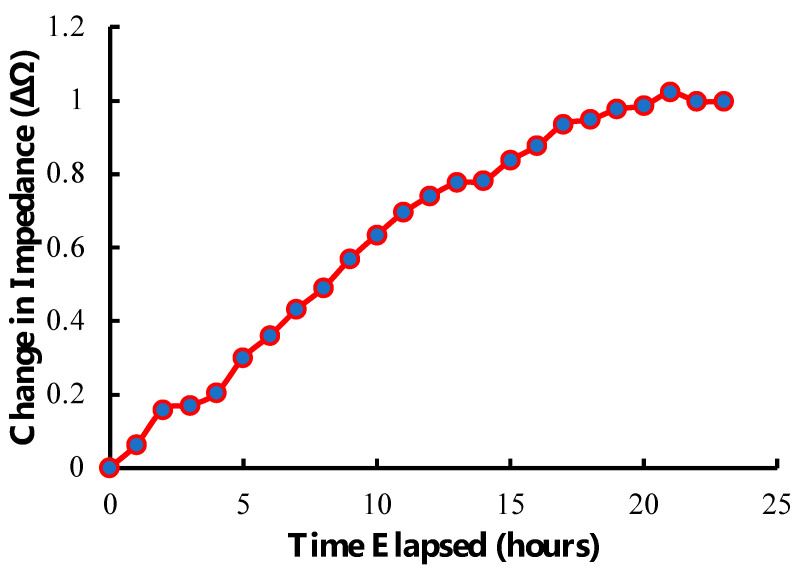
Illustration of the longitudinal tracking capability of the sensor and detection system showing the change in impedance of the sensor over time after seeding at 40,000 cells/cm^2^. The data displayed are a rolling average of 3 points.

Our detection system was capable of longitudinally tracking hMSCs seeded onto the sensor. As depicted in Figure 10, a steady rise in sensor response corresponded to the loading of cells, their increasing mechanical attachment to the surface, generation of extra-cellular matrix, and continued proliferation. As cells approached confluence on the sensor surface, the rate of change began to plateau. This behavior highlights the value of this approach for monitoring cell culture, as this technology can track cell growth in real time, non-invasively, and remotely. This technology also has the potential to indicate conditions of interest in cell manufacturing, such as cell attachment and confluence, by correlating acute changes in the slope of the longitudinal plot with the observed onset of those conditions.

### 3.4. Gelatin Surface Treatment

**Figure 11:**
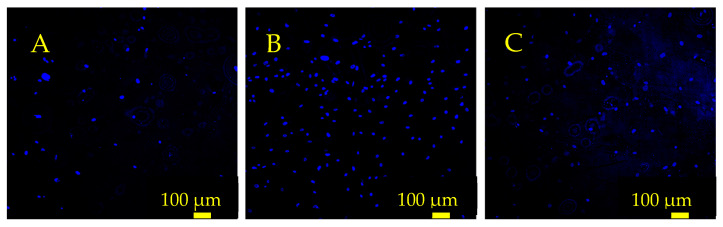
Fluorescence microscope images depicting hMSC nuclei on the sensor surface 24 h after seeding. (**A**) The control sensor that was not coated with gelatin. (**B**) A sensor that was soaked in gelatin solution for 30 min. (**C**) A sensor that was soaked in gelatin solution for 60 min. These sensors were seeded at 12,500 cells/cm^2^, and the cells were stained with Hoescht 33342 and imaged with a DAPI filter.

**Figure 12:**
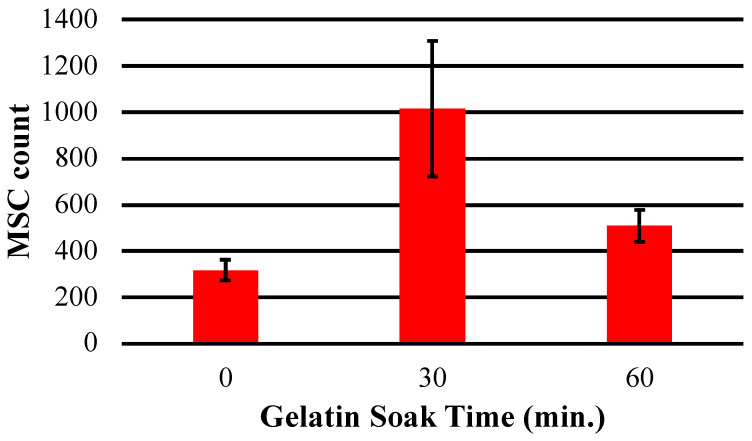
This chart depicts the average total number of hMSCs counted on the surface of sensors soaked in gelatin solution for 0, 30 and 60 min.

Analysis of cell number following gelatin surface treatment indicated that a conformal gelatin coating on the sensor surface had a positive effect on cell adhesion and proliferation (Figure 11 and Figure 12). Both the 30 and 60 min soak durations resulted in an increased number of cells attached compared to the control sensor that was not soaked in gelatin. Furthermore, the results indicated that the conformal gelatin layer can have a reverse effect depending on how long the sensor is soaked. The sensors that were soaked for 60 min resulted in fewer cells adhered to the surface compared to the sensors soaked for 30 min. It is well understood that the stiffness of a substrate can influence adherent cell behavior and proliferation [31,32,33,34]. More specifically, Rowlands et. al. concluded that MSCs grown on hydrogels of varying stiffness proliferated more as the stiffness of the hydrogel increased, with the stiffest surfaces having a reverse effect [35]. The results of this experiment agree with this conclusion: sensors treated with the 30 min soak hosted nearly three times as many cells as the control sensors and twice as many cells as the sensors that were soaked for 60 min.

### 3.5. Effects of Sensor Activity on Cell Morphology

**Figure 13:**
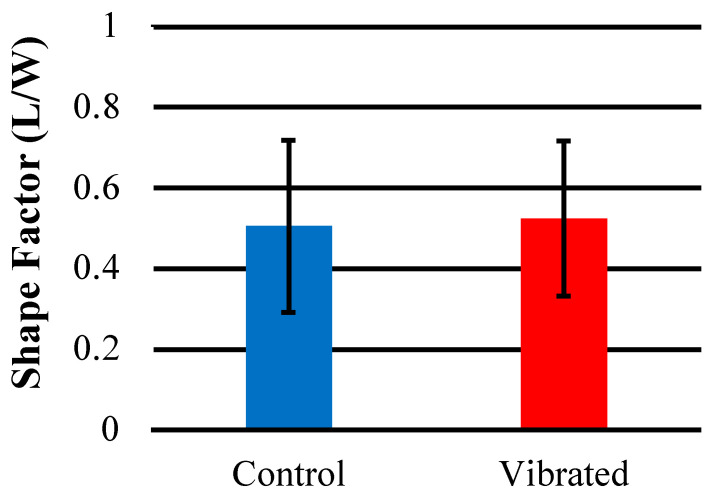
The average shape factor (L/W) of hMSCs imaged on the surface of sensors stained with Cell Tracker GreenTM and imaged with FITC filter (*n* = 10).

**Figure 14:**
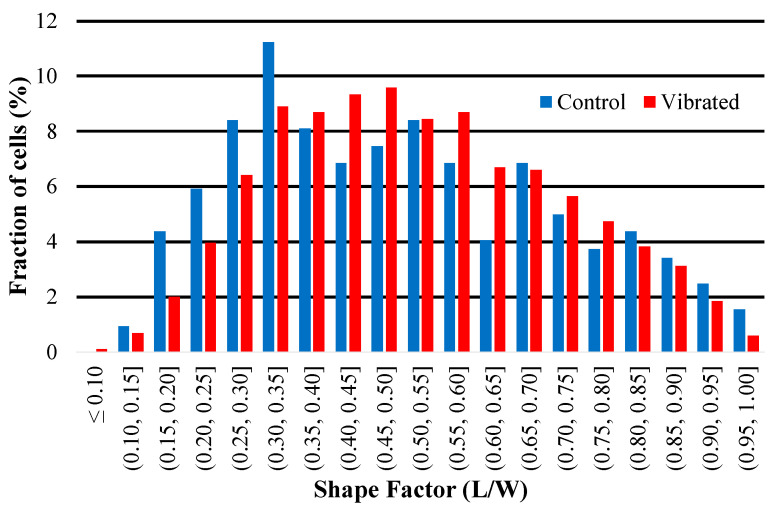
This histogram illustrates the distribution of shape factor (L/W) of hMSCs imaged on the surface of sensors. Skewness (control) = 0.35; skewness (vibrated) = 0.25.

**Figure 15:**
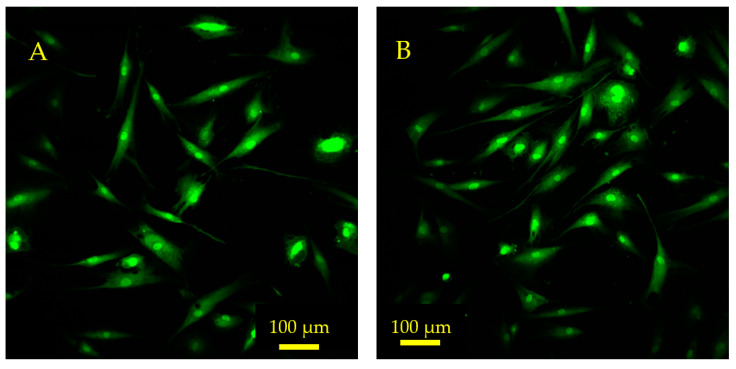
Fluorescence microscope images depict hMSCs growing on the sensor surface 24 h after seeding. (**A**) The control sensor that was not exposed to magnetic or mechanical stimulation. (**B**) An experimental sensor that was exposed to magnetic fields and mechanical resonance via the monitoring system. These sensors were seeded at 10,000 cells/cm^2^. The cells were stained with CellTrackerTM Green (CMFDA) dye prior to seeding and imaged using a FITC filter.

The results of a quantitative shape factor analysis (Figure 13 and Figure 14) indicate that there were no significant differences in the average shape factor between cells grown on sensors that were subjected to magnetic and mechanical stimulation and cells that were grown on a sensor without stimulation. Figure 14 shows regular distribution patterns with similar positive skewness. A qualitative visual inspection of fluorescent microscope images (Figure 15) confirms that the quality and morphology of the cells are the same or very similar.

## 4. Conclusions

The results indicate that the magnetoelastic sensors and monitoring system are a viable approach for tracking adherent cell growth remotely and non-invasively. The monitoring system was shown to be capable of longitudinally tracking cell growth, and a decreasing rate of change in sensor response over time could be observed as cell-loading approached confluence. Furthermore, examining the shape factor profiles of the vibrated and non-vibrated cells suggests that this technology can be used without affecting the quality and phenotype of the cells produced. A potential application of this monitoring system is to automate feedback-controlled processes for cell manufacturing. Continued development of this technology will focus on the adaptation of the monitoring system for classic stirred-tank bioreactor systems by adjusting the detection system and stimulus conditions for a larger volume reactor. To accommodate different bioreactor designs, the sensors and detection system will be fabricated in a variety of shapes and sizes. To translate this technology into industrial and clinical spaces, further investigation into the effects of magnetic fields and mechanical stimulation (generated by the monitoring system) on cell phenotype and quality will be needed. Additional work to miniaturize the sensors, optimize the detection system, and improve the surface conditions of the sensor for cell adhesion and proliferation will play a critical role in facilitating the integration of this technology into bioreactors of varying scale, design, and application.

## Data Availability

Not applicable.

## References

[1] Pereira Chilima T.D., Moncaubeig F., Farid S.S. (2018). Impact of allogeneic stem cell manufacturing decisions on cost of goods, process robustness and reimbursement. Biochem. Eng. J..

[2] Martin C., Olmos E., Collignon M., De Isla N., Blanchard F., Chevalot I., Marc A., Guedon E. (2017). Revisiting MSC expansion from critical quality attributes to critical culture process parameters. Process. Biochem..

[3] Deskins D.L., Bastakoty D., Saraswati S., Shinar A., Holt G.E., Young P.P. (2013). Human Mesenchymal Stromal Cells: Identifying Assays to Predict Potency for Therapeutic Selection. Stem. Cells Transl. Med..

[4] Shahdadfar A., Frønsdal K., Haug T., Reinholt F.P., Brinchmann J.E. (2005). In Vitro Expansion of Human Mesenchymal Stem Cells: Choice of Serum Is a Determinant of Cell Proliferation, Differentiation, Gene Expression, and Transcriptome Stability. Stem Cells.

[5] Krampera M., Galipeau J., Shi Y., Tarte K., Sensebe L. (2013). Immunological characterization of multipotent mesenchymal stromal cells—The International Society for Cellular Therapy (ISCT) working proposal. Cytotherapy.

[6] Justice C., Leber J., Freimark D., Pino Grace P., Kraume M., Czermak P. (2011). Online- and offline- monitoring of stem cell expansion on microcarrier. Cytotechnology.

[7] Arias L.R., Perry C.A., Yang L. (2010). Real-time electrical impedance detection of cellular activities of oral cancer cells. Biosens. Bioelectron..

[8] Kho D., Macdonald C., Johnson R., Unsworth C., O′Carroll S., Mez E., Angel C., Graham E. (2015). Application of xCELLigence RTCA Biosensor Technology for Revealing the Profile and Window of Drug Responsiveness in Real Time. Biosensors.

[9] Odeleye A.O.O., Castillo-Avila S., Boon M., Martin H., Coopman K. (2017). Development of an optical system for the non-invasive tracking of stem cell growth on microcarriers. Biotechnol. Bioeng..

[10] Pacella N., DeRouin A., Pereles B., Ong K.G. (2015). Geometrical modification of magnetoelastic sensors to enhance sensitivity. Smart Mater. Struct..

[11] Grimes C., Mungle C., Zeng K., Jain M., Dreschel W., Paulose M., Ong K. (2002). Wireless Magnetoelastic Resonance Sensors: A Critical Review. Sensors.

[12] Ruan C., Zeng K., Varghese O.K., Grimes C.A. (2003). Magnetoelastic Immunosensors: Amplified Mass Immunosorbent Assay for Detection of EscherichiacoliO157:H7. Anal. Chem..

[13] Possan A.L., Menti C., Beltrami M., Santos A.D., Roesch-Ely M., Missell F.P. (2016). Effect of surface roughness on performance of magnetoelastic biosensors for the detection of *Escherichia coli*. Mater. Sci. Eng. C.

[14] Ong K.G., Zeng K., Yang X., Shankar K., Ruan C., Grimes C.A. (2006). Quantification of multiple bioagents with wireless, remote-query magnetoelastic microsensors. IEEE Sens. J..

[15] Ong K.G., Leland J.M., Zeng K., Barrett G., Zourob M., Grimes C.A. (2006). A rapid highly-sensitive endotoxin detection system. Biosens. Bioelectron..

[16] Menti C., Henriques J.A.P., Missell F.P., Roesch-Ely M. (2016). Antibody-based magneto-elastic biosensors: Potential devices for detection of pathogens and associated toxins. Appl. Microbiol. Biotechnol..

[17] Huang S., Yang H., Lakshmanan R.S., Johnson M.L., Wan J., Chen I.-H., Wikle H.C., Petrenko V.A., Barbaree J.M., Chin B.A. (2009). Sequential detection of Salmonella typhimurium and Bacillus anthracis spores using magnetoelastic biosensors. Biosens. Bioelectron..

[18] Guntupalli R., Hu J., Lakshmanan R.S., Huang T.S., Barbaree J.M., Chin B.A. (2007). A magnetoelastic resonance biosensor immobilized with polyclonal antibody for the detection of Salmonella typhimurium. Biosens. Bioelectron..

[19] Guntupalli R., Lakshmanan R.S., Hu J., Huang T.S., Barbaree J.M., Vodyanoy V., Chin B.A. (2007). Rapid and sensitive magnetoelastic biosensors for the detection of Salmonella typhimurium in a mixed microbial population. J. Microbiol. Methods.

[20] Pang P., Huang S., Cai Q., Yao S., Zeng K., Grimes C.A. (2007). Detection of Pseudomonas aeruginosa using a wireless magnetoelastic sensing device. Biosens. Bioelectron..

[21] Xiao X., Guo M., Li Q., Cai Q., Yao S., Grimes C.A. (2008). In-situ monitoring of breast cancer cell (MCF-7) growth and quantification of the cytotoxicity of anticancer drugs fluorouracil and cisplatin. Biosens. Bioelectron..

[22] Holmes H.R., Tan E.L., Ong K.G., Rajachar R.M. (2012). Fabrication of Biocompatible, Vibrational Magnetoelastic Materials for Controlling Cellular Adhesion. Biosensors.

[23] Trierweiler S., Holmes H., Pereles B., Rajachar R., Ong K.G. (2013). Remotely activated, vibrational magnetoelastic array system for controlling cell adhesion. J. Biomed. Sci. Eng..

[24] Holmes H.R., DeRouin A., Wright S., Riedemann T.M., Lograsso T.A., Rajachar R.M., Ong K.G. (2014). Biodegradation and biocompatibility of mechanically active magnetoelastic materials. Smart Mater. Struct..

[25] Meyers K.M., Ong K.G. (2021). Magnetoelastic Materials for Monitoring and Controlling Cells and Tissues. Sustainability.

[26] Shekhar S., Karipott S.S., Guldberg R.E., Ong K.G. (2021). Magnetoelastic Sensors for Real-Time Tracking of Cell Growth. Biotechnol. Bioeng..

[27] PRNewswire Mesenchymal Stem Cells Market Size Worth $6.1 Billion By 2028: Grand View Research, Inc. https://www.prnewswire.co.uk/news-releases/mesenchymal-stem-cells-market-size-worth-6-1-billion-by-2028-grand-view-research-inc--873119294.html.

[28] Metglas^®^ 2826MB. www.metglas.com.

[29] Skinner W.S., Zhang S., Guldberg R.E., Ong K.G. (2022). Magnetoelastic Sensor Optimization for Improving Mass Monitoring. Sensors.

[30] Neuhuber B., Swanger S.A., Howard L., Mackay A., Fischer I. (2008). Effects of plating density and culture time on bone marrow stromal cell characteristics. Exp. Hematol..

[31] Gerardo H., Lima A., Carvalho J., Ramos J.R.D., Couceiro S., Travasso R.D.M., Pires Das Neves R., Grãos M. (2019). Soft culture substrates favor stem-like cellular phenotype and facilitate reprogramming of human mesenchymal stem/stromal cells (hMSCs) through mechanotransduction. Sci. Rep..

[32] Wolf K., Te Lindert M., Krause M., Alexander S., Te Riet J., Willis A.L., Hoffman R.M., Figdor C.G., Weiss S.J., Friedl P. (2013). Physical limits of cell migration: Control by ECM space and nuclear deformation and tuning by proteolysis and traction force. J. Cell Biol..

[33] Ramos J.R.D., Travasso R., Carvalho J. (2018). Capillary network formation from dispersed endothelial cells: Influence of cell traction, cell adhesion, and extracellular matrix rigidity. Phys. Rev. E.

[34] Rens E.G., Merks R.M.H. (2017). Cell Contractility Facilitates Alignment of Cells and Tissues to Static Uniaxial Stretch. Biophys. J..

[35] Rowlands A.S., George P.A., Cooper-White J.J. (2008). Directing osteogenic and myogenic differentiation of MSCs: Interplay of stiffness and adhesive ligand presentation. Am. Physiol. Soc..

